# Unexpected Clinical Status of Phyllodes Tumor of the Breast: Massive Bleeding

**DOI:** 10.7759/cureus.65923

**Published:** 2024-08-01

**Authors:** Alp Ömer Cantürk, Hasan Yilmaz, Buse Yildirim, Saime Gul Barut, Fazilet Erözgen

**Affiliations:** 1 Surgery, University of Health Sciences, Haseki Training and Research Hospital, Istanbul, TUR; 2 Pathology, Haseki Training and Research Hospital, Istanbul, TUR

**Keywords:** breast tumor, emergency breast surgery, blood transfusion, massive bleeding, giant bilateral phyllodes tumor

## Abstract

Regardless of whether they are benign or malignant, phyllodes tumors can behave unpredictably and aggressively. Sometimes they grow so quickly and can cause clinically different, rare, and difficult situations to manage. Because of these characteristics, they can be fatal, even if they are benign. Sometimes, emergency surgical operations may be required due to the occurrence of these conditions even before the diagnosis. We report the first case of a massive bilateral phyllodes tumor, which causes severe bleeding because of rapid growth, which resulted in emergency surgery performed after a blood transfusion. The pathological diagnosis had not yet been confirmed at the time we operated on the patient, and the postoperative pathologic examination revealed that it was a phyllodes tumor. After the successful surgical operation, the patient recovered and was discharged. In this case report, we shared the presentation and management of the emergency phyllodes tumorous phenomenon. We also conveyed our views on what could have been done differently in the management of the presented case.

## Introduction

Phyllodes tumors are rare diseases that can be benign or malignant and may cause different clinical findings [1]. Some may exhibit clinical manifestations such as benign fibroadenoma, while others may lead to bleeding or even death due to rapid growth. Others may metastasize to distant organs [2]. Since it was first described by Müller in 1838, it has been referred to by many different terminological names [3]. Phyllodes tumors, which constitute 1% of breast cancers, usually occur in women in their forties [3,4]. Treatment is extensive surgical excision [5]. In some cases, it may be necessary to plan surgery before the diagnosis can be made. Breast rupture associated with phyllodes tumors was reported in the literature, too [6]. We presented a case in which surgical intervention had been necessary before the diagnosis could have been made because of severe bleeding.

## Case presentation

**Figure 1:**
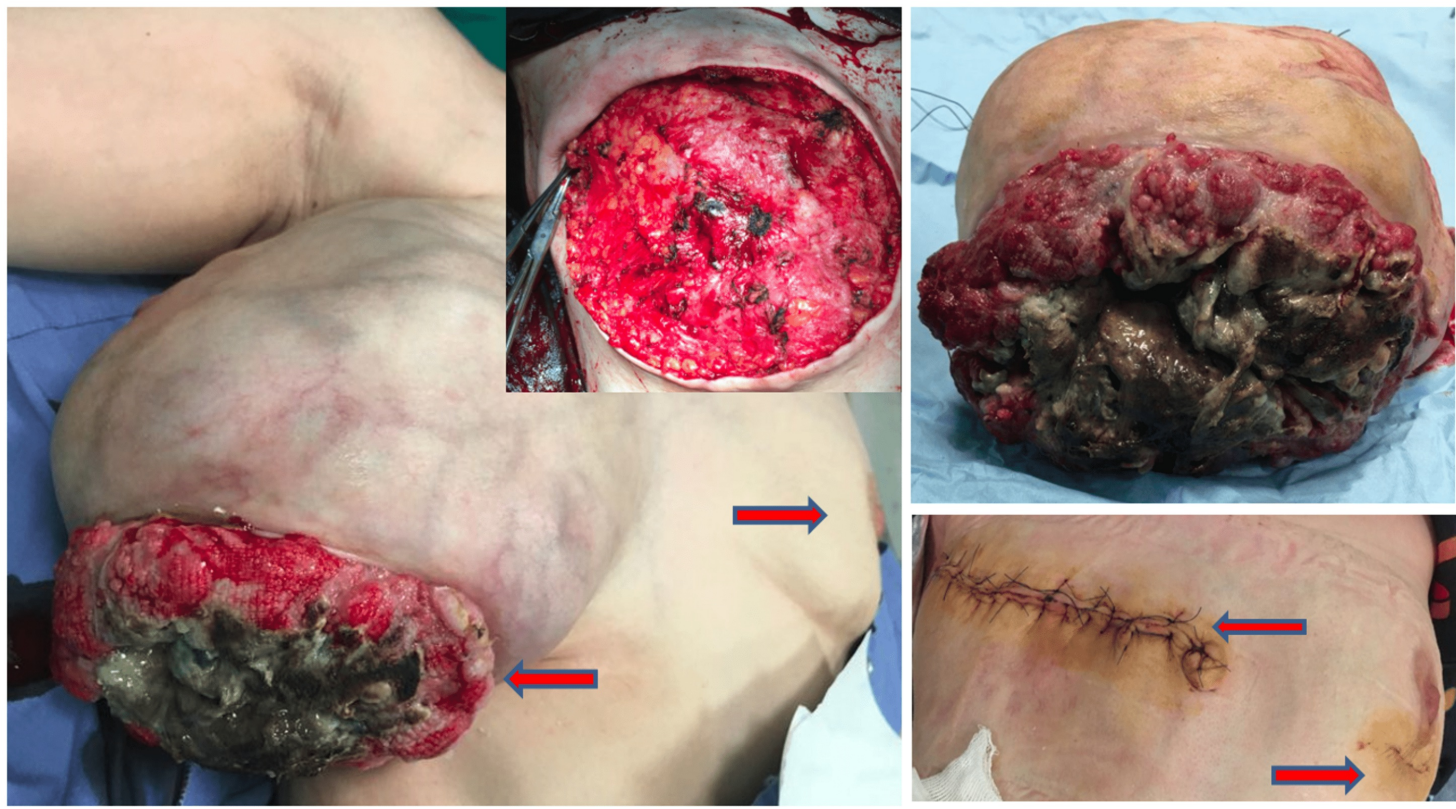
Surgical images of bilateral phyllodes tumor

**Figure 2:**
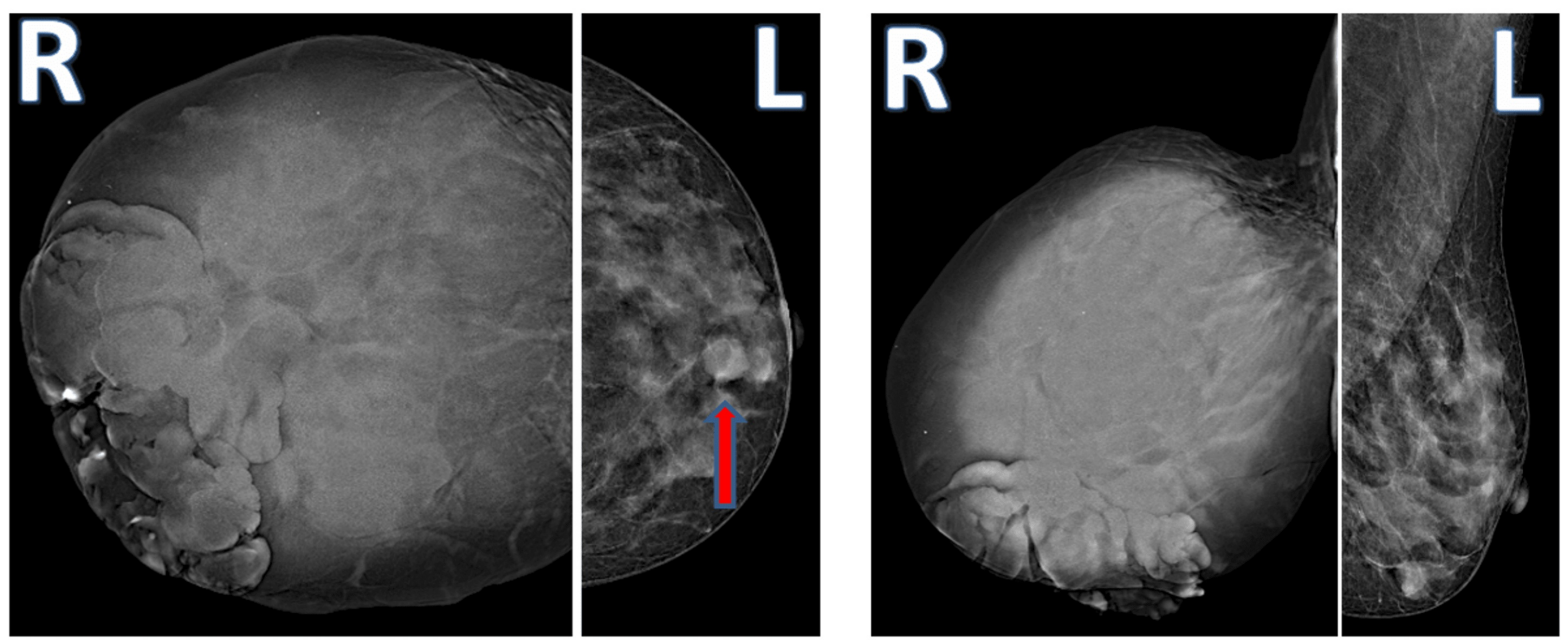
Right and left mammographic images of bilateral phyllodes tumor

**Figure 3:**
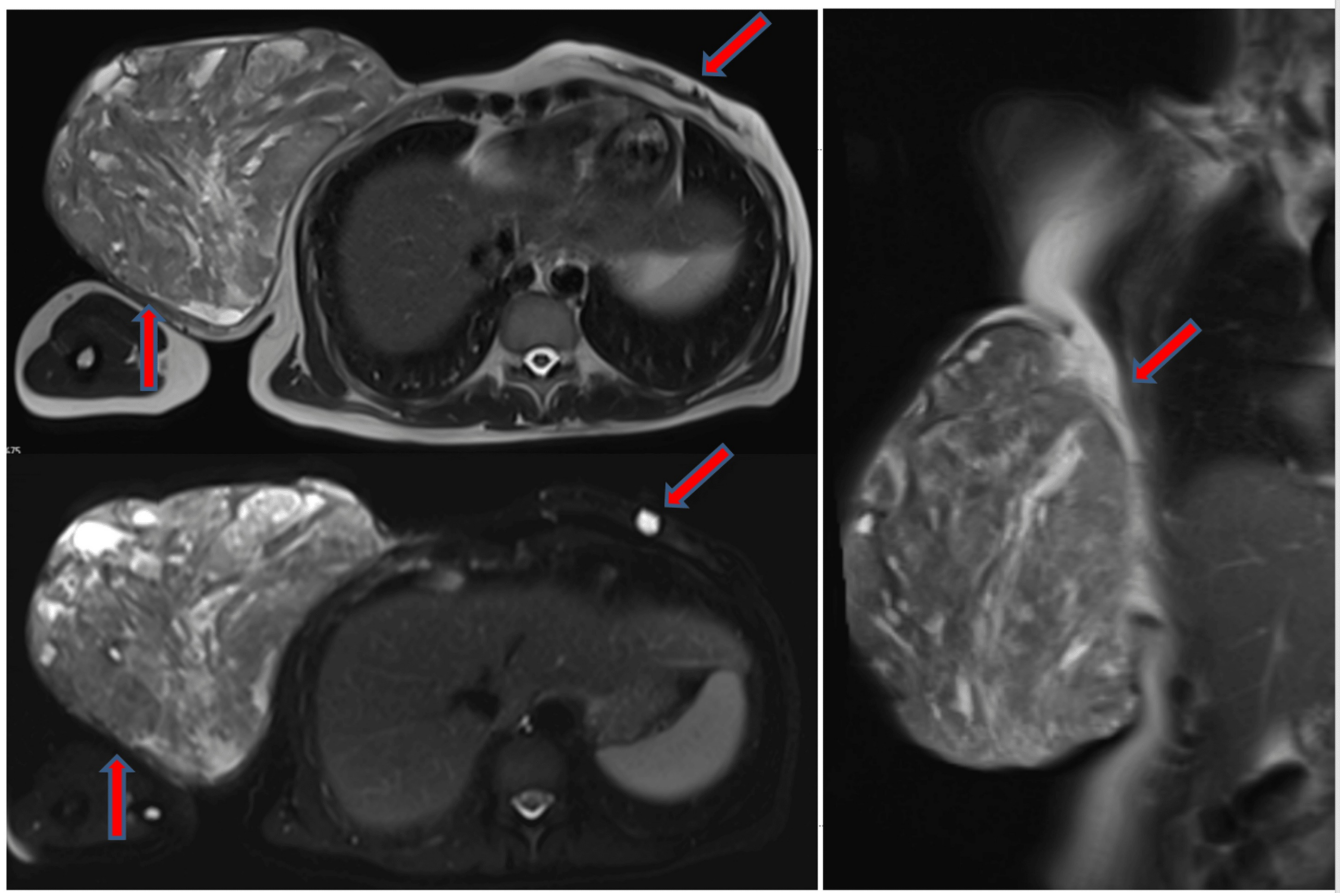
Right and left MRI images of bilateral phyllodes tumor

**Figure 4:**
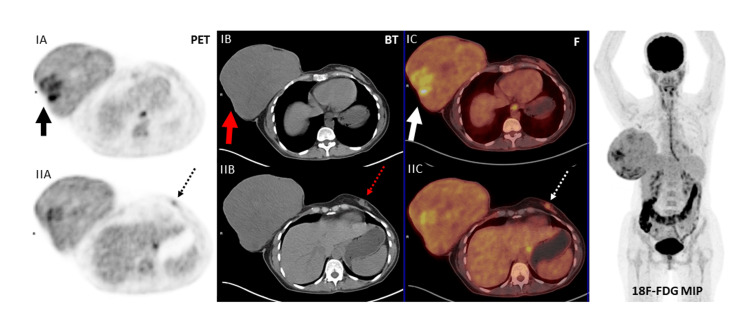
Preoperative PET-CT images of bilateral phyllodes tumor

**Figure 5:**
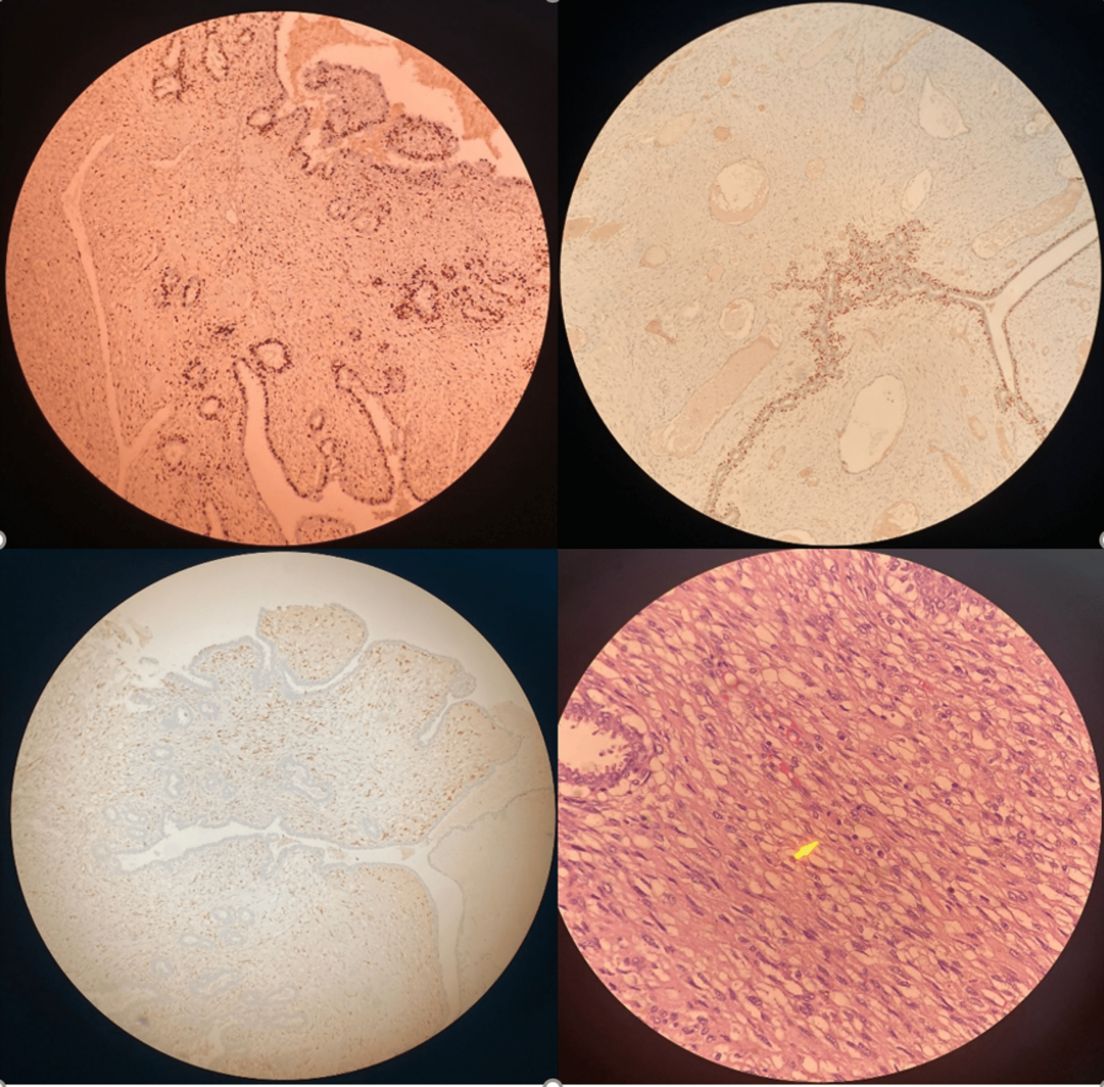
Postoperative pathology

A 41-year-old female patient presented with the complaint of a rapidly growing mass in her right breast in the last six months. The patient had never consulted a doctor before because of this complaint. She stated that she did not seek medical advice because she expected the mass to vanish by itself, but she could no longer stand because of pain and bleeding. She had no family history of breast cancer. On physical examination, there was a 25 cm mass in the right breast that completely covered the breast, which was ulcerated, necrotic, and prone to bleeding (Figure 1). This lesion completely covered the whole breast and the nipple had disappeared due to the mass. A second mass of 1 cm was also detected in the upper quadrant of the left breast. Since the patient did not have any definitive diagnosis, routine imaging, and pathological evaluations were planned for the patient. Mammographic and ultrasonographic imaging revealed a 25 cm BI-RADS 5 mass completely filling the right breast, with the interval between the mass and the skin lost. There was a second mass of 1 cm in the left breast (Figure 2). Pathological samples were taken from both masses, and the results could not be distinguished between fibroadenoma and phyllodes tumor. Surgical excision was planned for the patient, but since a definitive diagnosis could not be made, MR and PET-CT imaging were planned prior to surgery. MRI results were similar to USG, with additional suspicious lymph nodes in the right axilla (Figure 3). PET-CT identified the mass in the right breast as a primary malignant lesion (SUV max: 10.35). It defined the lesion in the left breast as a second mass, which was a metastasis of the mass in the right breast (SUV max: 10.35). No pathological lymph nodes were detected in either axillary region (Figure 4). After these results, the patient was hospitalized, and a biopsy was planned again. Surgery was then planned because there was still no definitive diagnosis. Without having the opportunity to do all this, severe bleeding occurred from the mass in the right breast, and it did not stop with compression and adrenaline dressing. A blood transfusion was planned for the patient. Despite the transfusion, the patient's hemoglobin was severely reduced. After the patient's hemoglobin value fell to 5.1, the plan was changed, and the patient was taken for emergency surgery; a simultaneous blood transfusion was planned. The patient underwent right right-modified radical mastectomy. The mass in the left breast was also removed by extensive local excision (Figure 1). On pathological examination, hypercellular stroma, epithelial cells with cystic openings, nuclear pleomorphism, and low mitotic activity were consistent with malignant phyllodes tumor (Figure 5). The lesion in the left breast was evaluated as metastasis. The patient was discharged without complications. The patient was followed up with regular intervals for three years and no recurrence or metastasis was detected.

## Discussion

Phyllodes tumor is an unpredictable and aggressive disease that accounts for 0.3-1% of breast malignancies [7]. Whether phyllodes tumors are benign or malignant, their rapid growth can always increase the severity of the clinical condition. The tumor in the presented case measures 25 cm in diameter, which is significantly larger than the majority of cases reported in the literature. The significant tumor size of our case was because of the negligence of the patient. If the patient did not have complaints of breast pain and bleeding in the breast, she might not have consulted a physician again. Here, the patient's negligence and level of education were essential as they also affected her morbidity. In this case, the development of a metastatic lesion to the other breast may be because the patient has not consulted the doctor. The diagnosis of phyllodes tumor is made by radiological and pathological evaluations. Due to inadequate diagnostic assessment during the patient's first admission, the management and surgical process were prolonged. Perhaps this is why the patient's lesion has progressed, and the patient who presents with bleeding needs a transfusion and emergency surgery. Planning MRI and PET-CT imaging during the treatment process may have caused the process to be a little longer. All of this may have led to the need for urgent surgery before the patient's diagnosis was confirmed. Modified radical mastectomy was performed on the right breast, and extensive local excision was performed on the left breast. If there was no urgent need for surgery, radiotherapy could have been planned for the patient before surgery. There are articles in the literature that suggest preoperative radiotherapy reduces local recurrence [8]. We have been following our patient for three years and we have not detected any findings in favor of local recurrence, which may be due to the wide surgical excision. In such cases, the treatment management of the patient should be applied in a personalized way. Optimal response to treatment can be achieved by making patient-based decisions.

## Conclusions

At the stage of diagnosis and treatment of phyllodes tumors, it should be borne in mind that although benign, they can overgrow, bleed, and lead to severe morbidities. Consequently, it should be known that these tumors are aggressive in all respects. Treatment management should be evaluated on a patient-by-patient basis, morbidities should be considered when necessary, and it should be kept in mind that sometimes emergency surgical operations may need to be planned even if the diagnosis is not confirmed. The case of massive bilateral phyllodes tumor and emergency surgery due to the need for transfusion is first described by us in the literature. We aimed to contribute to the literature by presenting this valuable and unique phenomenon.
